# Supplementary material for: WordCommentsAnalyzer: A windows software tool for qualitative research
Source: F1000Res. 2018 Sep 4;7:536. Originally published 2018 May 3. [Version 2] doi: 10.12688/f1000research.14819.2 (PMC6137411; doi:10.12688/f1000research.14819.2)
Supplement: Supplementary file 3 [file f1000research-7-17614-s0002.tgz › b2a79496-4ada-47c4-9682-a99ce867836d.zip!/Eight Iranian Health Journals/Journal of Knowledge and Health_33.docx]

© Journal of Knowledge and Health. This work is licensed under CC BY 4.0 (https://creativecommons.org/licenses/by/4.0/).

Volume & Issue: دوره 5، شماره 1: 1389

Title: بررسی میزان پاسخ سیستم ایمنی بیماران HIV/AIDS به واکسیناسیون هپاتیت B

http://knh.shmu.ac.ir/index.php/site/article/view/126

مقدمه: با توجه به احتمال بالاتر ازمان عفونت هپاتیت B در افراد دارای عفونت HIV‌، ایمن­سازی بر علیه هپاتیت B در این بیماران ضرورت دارد. هدف از این مطالعه بررسی عوامل مؤثر بر پاسخ یا عدم پاسخ به واکسیناسیون هپاتیت B در موارد ایرانی HIV+ در مقایسه با گروه شاهد سالم بوده است. مواد و روش­ها: از اردیبهشت ماه 86 تا خرداد ماه 87 ,50 مورد HIV+ و 50 نفر افراد گروه شاهد سالم که از نظر شواهد سرولوژیک هپاتیت B منفی بودند، تحت واکسیناسیون هپاتیت B در سه دز اولين مراجعه، 1 و 6 ماه بعد قرار گرفتند و 2-1 ماه بعد از تزریق آخرین دز واکسن نسبت به تعیین تیتراژ HBS Ab آن­ها اقدام و میزان پاسخ به واکسن بر اساس تیتر HBS Ab بررسی گردید. نتایج: در گروه HIV+ تعداد 40 نفر (80%) و در گروه شاهد 46 نفر (92%) به واکسن پاسخ دادند ولی اختلاف بین دو گروه از نظر آماری معنادار نبود (8/0=P). در گروه HIV+ پاسخ به واکسن با سن، جنس، BMI (شاخص توده بدنی)، مصرف سیگار، مصرف الکل، راه ابتلا، مرحله بیماری و درمان ضد ويروس عامل ايدز ارتباط نداشت. میزان پاسخ در گروه HIV+، ارتباط معناداری با سطح CD4 بیماران داشت (03/0=P). نتیجه­گیری: واکسیناسیون هپاتیت B در بیماران HIV+ بهتر است در اوایل بیماری و با همان دز معمول صورت گیرد و در بیماران با CD4<200 cell/µl تیتر HBS Ab در فواصل معین اندازه­گیری شود.

Title: بررسی شاخص¬های استرس اکسیداتیو در زایمان طبیعی و ارتباط آن با شاخص¬های استرس اکسیداتیو در نوزاد

http://knh.shmu.ac.ir/index.php/site/article/view/134

مقدمه:. پراکسیداسیون لیپید یک پدیده طبیعی است که به­طور مداوم در سطوح پایین در بدن تولید می‌شود با این حال اگر توسط دفاع آنتی­اکسیدانی کنترل نشوند، این واکنش‌های پراکسیداسیونی می­تواند برای سلول‌ها و غشای آن‌ها سمی باشد. به­نظر می­رسد حاملگی و زایمان با استرس اکسیداتیو همراه باشد. لذا این تحقیق با هدف بررسی شاخص­های استرس اکسیداتیو در بارداری و زایمان انجام شده است. مواد و روش­ها: در اين مطالعه مقطعي دو گروه 60 نفره از زنان باردار و زنان زايمان كرده انتخاب و پس از تكميل فرم رضايت آگاهانه، cc5 خون جهت محاسبه شاخص­هاي اكسيداتيو از آنان گرفته شد. در گروه زنان باردار در هفته 40- 38 بارداري و در گروه دوم در اتاق زايمان، در طي مرحله دوم زايمان و ديلاتاسيون كامل خونگيري انجام شده است. معيارهاي ورود شامل: عدم عارضه حاد و مزمن حاملگي، زايمان اول و حاملگي تك قلو بود. بلافاصله پس از خروج جنین و مراقبت­های اولیه، از خون بند ناف نوزادان مادران زایمان کرده cc5 خون گرفته  شد. نتایج: میانگین سنی در گروه زنان باردار 52/0± 8/22 سال و در گروه دوم 8/1± 4/21 سال مي­باشد. در اين مطالعه ميانگين لیپیدپراکسیدایون زمان زايمان (nmol/l 58/5) به­طور معناداري بيش­تر از دوران حاملگي (nmol/l 64/3) بوده و هم­چنين بين مقادير لیپیدپراکسیدایون گروه­هاي تيول و FRAP مادران با زايمان طبيعي و نوزادان همبستگي معنادار و مستقيمي وجود دارد. نتیجه­گیری: نتایج نشان داد که اختلاف آماری معناداری بین مقادیر پر اکسیداسیو لیپید در حاملگی و زایمان وجود دارد به­طوری که پر اکسیداسیو لیپید در زایمان افزایش یافته است. از طرفی بین شاخص­های استرس اکسیداتیو زایمان با مقادیر آن در خون نوزاد ارتباط معنادار وجود داشت. به نظر می­رسد تقویت دفاع آنتی­اکسیدان­های مادران ضروری بوده و استفاده مادران از مکمل­های آنتی­اکسیدان در بارداری مفید باشد.

Title: بررسي عوامل مرتبط با رفتارهاي پيشگيري¬کننده سرطان پوست در دانش¬آموزان دختر دبيرستاني شهر يزد بر اساس تئوري انگيزش محافظت در سال 88- 1387

http://knh.shmu.ac.ir/index.php/site/article/view/135

مقدمه: سرطان پوست شايع­ترين نوع سرطان در شهر يزد مي­باشد و دوران کودکي و نوجواني يک زمان مهم و ويژه براي پيشگيري از سرطان پوست در آينده است. هدف مطالعه حاضر تعيين عوامل مرتبط با انجام رفتارهاي پيشگيري­کننده سرطان پوست در دانش­آموزان دختر دبيرستاني شهر يزد با استفاده از تئوري انگيزش محافظت است. مواد و روش­ها: شرکت­کننده­هاي اين مطالعه مقطعي، شامل 360 دانش­آموز دختر مقطع دبيرستان از 4 دبيرستان شهر يزد بودند. داده­ها از طريق پرسش­نامه خود ایفا جمع­آوري گرديد. داده­هابا استفاده از نرم­افزار آماري SPSS و تست­هاي آماري ANOVA T-test, ضريب همبستگي پيرسون و رگرسيون مورد تجزيه و تحليل قرار گرفتند. نتايج: ضريب همبستگي پيرسون نشان داد که بين رفتارهاي محافظت­کننده سرطان پوست با سازه­هاي حساسيت درک شده، شدت درک شده، ترس، خودکارآمدي، کارآمدي پاسخ و ارزيابي کنار آمدن، همبستگي مثبت و با پاداش، هزينه­هاي پاسخ و ارزيابي تهديد، همبستگي منفي در سطح 01/0 وجود داشت. ميزان پيشگويي انگيزش محافظت توسط دو فرآيند ميانجي شناختي ارزيابي تهديد و ارزيابي کنار آمدن، 54% بود که در اين ميان نقش ارزيابي تهديد (335/0=β) بيش­تر بود. هم­چنين ميزان پيشگويي رفتارهاي محافظت­کننده به­وسيله سازه انگيزش محافظت 41% بود.نتيجه­گيري: نتايج اين مطالعه اثربخشي تئوري انگيزش محافظت را در پيشگويي رفتارهاي پيشگيري­کننده از سرطان پوست در شرکت­کنندگان تأييد مي­کند

Title: تأثير طب فشاري نقطه LI4 بر طول مراحل زايمان، شاخص¬هاي فيزيولوژيک مادر و نمره آپگار نوزاد

http://knh.shmu.ac.ir/index.php/site/article/view/138

مقدمه: زايمان طولاني با زايمان سزارين و کاهش pH بندناف همراه است. همچنین خونريزي و عفونت که با زايمان طولاني همراه هستند، از علل منتهي به مرگ مادر محسوب مي­شوند. به­دليل اهميت طول زايمان اين مطالعه تأثير طب فشاري نقطه LI4 را بر طول مراحل زايمان، شاخص­هاي فيزيولوژيک مادر و نمره آپگار نوزاد مورد بررسي قرار مي­دهد. مواد و روش­ها: اين مطالعه کارآزمايي باليني تصادفي با گروه شاهد بر روي 100 زن باردار ترم كه در فاز فعال مرحله اول زايمان(اتساع 5-3 سانتي­متر) بوده و به مركز آموزشي درماني فاطميه شهرستان شاهرود مراجعه کردند، انجام شده است. نمونه­ها به­طور تصادفي به دو گروه آزمايش (طب فشاري 50 نفر) و شاهد (لمس50 نفر) تقسيم شدند. گروه آزمايش، 20 دقيقه طب فشاري در نقطهLI4  در هر دو دست به­طور همزمان و گروه شاهد به همان طريق لمس در همان نقطه را بدون هيچ فشاري دريافت نمودند.  نتايج: نتايج مطالعه نشان داد که طول مراحل اول و دوم زايمان در گروه طب فشاري به­طور معناداري از گروه شاهد کم­تر است (038/0P= و 001/0P=). يافته­ها اختلاف آماري معناداري بين دو گروه را از نظر  شاخص­هاي فيزيولوژیک مادر (تعداد نبض و تنفس) نشان نداد. بين نمرات آپگار دقايق اول و پنجم نوزاد نيز اختلاف آماري معناداري بين دو گروه مشاهده نشد (3/0P= و 2/0P=).نتيجه­گيري: از يافته­هاي اين پژوهش مي­توان استنباط کرد که طب فشاري نقطه  LI4سبب کاهش طول مراحل اول و دوم زايمان شده و بر روي نبض و تنفس مادر و نمره آپگار نوزاد تأثيري ندارد. لذا استفاده از اين روش نوين، بي­خطر و ساده که نياز به صرف هيچ­گونه هزينه و ابزار ندارد، در مراکز زايمان توصيه مي­شود

Title: تأثير آموزش از طريق فيلم بر خستگي و كمردرد بيماران تحت آنژيوگرافي كرونر

http://knh.shmu.ac.ir/index.php/site/article/view/143

مقدمه: از آنجايي كه بيماران تحت آنژيوگرافي كرونر بايستي به­منظور جلوگيري از عوارض اين روش حداقل به­مدت 6 ساعت استراحت مطلق در تخت داشته باشند، اين بي­حركتي سبب خستگي و كمردرد در اين قبيل بيماران مي­شود. اين مطالعه با هدف تأثير آموزش از طريق فيلم بر خستگي و كمردرد بيماران تحت انجام آنژيوگرافي كرونر انجام شده است. مواد و روش­ها: در اين مطالعه نيمه تجربي، 128 بيمار كانديد آنژيوگرافي كرونر مراجعه‌کننده به بيمارستان بر طبق معيارهاي ورود و خروج انتخاب و به­طور تصادفي به دو گروه كنترل و آزمون اختصاص یافتند. بيماران گروه كنترل، آموزش شفاهی روتین توسط پرستاران بخش آنژیوگرافی را دریافت کردند و براي گروه آزمون يک فيلم آموزشي حاوي اقدامات لازم قبل، حين و بعد از آنژيوگرافي ارايه شد. بعد از انجام آنژيوگرافي ميزان خستگي و كمردرد بيماران دو گروه در 4 مرحله در طي 6 ساعت استراحت در تخت با استفاده از مقياس قابل قياس بصري (Visual Analogue Scale=VAS) سنجيده شد. تجزيه و تحليل داده‌ها با استفاده از روش‌هاي آماري توصيفي و استنباطي نظير آزمون تي مستقل و آنالیز واریانس با اندازه گیری­های تكراري و نرم‌افزار SPSS 15 انجام گرفت. نتايج: ميانگين نمره خستگي و كمردرد بيماران در مرحله اول اندازه­گيري بين دو گروه اختلاف معناداري را به لحاظ آماري نشان نداد (4/0=P). در مراحل ديگر اندازه­گيري، ميزان خستگي و كمردرد بيماران گروه كنترل به­طور معناداري بيش­تر از بيماران گروه آزمون بود (001/0P<). نتيجه‌گيري: نتايج اين مطالعه كاربرد آموزش از طريق فيلم را در كاهش ميزان خستگي و افزايش تحمل بيماران تحت آنژيوگرافي كرونر تأييد مي­كند. توصيه مي­شود از اين روش به­عنوان يكي از اجزاء برنامه آماده­سازي بيماران قبل از روش­های تهاجمي به­منظور افزايش رضايت­مندي و كاهش خستگي و كمردرد بيماران استفاده شود.

Title: بررسي اثر طب فشاري در نقطه liv3 بر شدت و مدت ديسمنوره اوليه

http://knh.shmu.ac.ir/index.php/site/article/view/148

مقدمه: ديسمنوره اوليه يکي از شايع‌ترين اختلالات در زنان است که سبب کاهش کارايي زنان مي‌گردد. طبق نظريات طب چيني درمان ديسمنوره، به تعديل انرژي و خون نياز دارد. هدف اين مطالعه بررسي اثر طب فشاري نقطه liv3 بر شدت و مدت ديسمنوره اوليه مي­باشد. مواد و روش­ها: در اين كارآزمايي باليني تعداد 194 نفر از دانشجويان مركز تربيت معلم نسيبه كه واجد شرايط ورود به مطالعه بودند انتخاب و در سيكل اول قاعدگي شدت و مدت درد تعيين و سپس افراد به­طور تصادفي به دو گروه مداخله و شاهد تقسيم شدند. در سيكل دوم، سوم و چهارم فشار در نقطه liv3 و فشار در نقطه پلاسبو، 3 تا 7 روز قبل از شروع قاعدگي، به مدت20 دقيقه در روز انجام گرديد. با شروع خونريزي در طي اين سه سيكل شدت و مدت ديسمنوره تعيين گرديد. ابزار گردآوري داده­ها صورتك­ هاي ونگ بيكر (براي سنجش درد)، پرسش­نامه بك، ساعت، دستگاه 900 Acuhealth tens pro و Force Guage بود. تجزيه و تحليل داده­ها با استفاده از آزمون c2، من ويتني، فريدمن و t مستقل انجام شد. نتايج: در سيكل اول از نظر شدت ديسمنوره بين گروه­ها تفاوتي وجود نداشت )40/0=P) ولي در سيكل دوم (05/0P<)، سوم (001/0P<) و چهارم (001/0P<) تفاوت معناداري بين دو گروه وجود داشت. ميانه شدت ديسمنوره در گروه مداخله و شاهد در سيكل اول برابر با 6 بود و در سيكل چهارم در گروه مداخله 4 و در گروه شاهد 6 بود. از نظر مدت درد در سيكل اول و دوم اختلاف معناداري بين گروه­ها وجود نداشت، ولي در سيكل سوم (05/0P<) و چهارم (001/0P<)، در گروه مداخله مدت درد به­طور معناداري نسبت به گروه كنترل كاهش يافته بود. نتيجه­گيري: در اين مطالعه استفاده از فشار در نقطه  liv3در کاهش طول مدت و شدت ديسمنوره اوليه مؤثر بوده است، لذا به­عنوان درماني كاربردي، مؤثر، ارزان و در دسترس جهت درمان ديسمنوره اوليه پيشنهاد مي­شود.

Title: تأثير كفي طبي بر ميزان مصرف انرژي افراد دونده داراي صافي كف پا

http://knh.shmu.ac.ir/index.php/site/article/view/151

مقدمه: کفی طبی به­عنوان یک درمان محافظتی در افراد دارای صافی کف پا استفاده می­شود. هدف از مطالعه حاضر بررسی تأثير كفي طبي بر مصرف انرژي 20 فرد دونده داراي صافي كف پا مي باشد. مواد و روش­ها: در اين مطالعه از يك تردميل و دستگاه Quark b2 جهت اندازه‌گيري مصرف اكسيژن استفاده شد. هم­چنين براي افراد يك جفت كفي طبي به اندازه پاي بيمار تهيه شد. در اين مطالعه بيشينه ضربان قلب، مصرف اكسيژن و بيشينه مصرف اكسيژن بيماران داراي صافي كف پا، با و بدون استفاده از كفي طبي اندازه‌گيري و محاسبه گرديد.داده­ها با استفاده از آزمون t جفت در دو حالت مقايسه شد. نتایج: در اين مطالعه ميانگين مصرف اكسي‍ژن (ميلي ليتر در دقيقه) از 6/4504 به 3/4488 و بيشينه اكسيژن مصرفي برحسب ميلي­ليتر در دقيقه به ازاي هر كيلوگرم وزن بدن از 6/63 به 4/63 و بيشينه تعداد ضربان قلب از 7/168 به 0/168 بعد از استفاده از كفي طبي كاهش يافته كه كاهش مشاهده در هر سه حالت معنادار مي­باشد (05/0P<). نتیجه­گیری: وسایل کمکی پا منجر به بهبود راستای مفاصل اندام تحتانی در بیماران دارای صافی کف پا می­شوند، بنابراین رابطه طول- تنشن عضلات بهبود می­یابد که این مسأله مانع از خستگی عضلات در دویدن­های با مسافت­های زیاد می­شود. در زمانی که از کفی طبی اندازه پای فرد برای بیماران استفاده شد، میزان مصرف انرژی آن­ها در حین دویدن کاهش پیدا کرد.

Title: بررسي عوامل ميكروبي در استفاده¬كنندگان روش¬هاي هورموني و جراحي پيشگيري از بارداري

http://knh.shmu.ac.ir/index.php/site/article/view/155

مقدمه: واژينيت از شایع­ترین تشخیص­های ژنیکولوژیک است که سالانه درصد قابل توجهی از ویزیت­های سرپایی را به خود اختصاص می­دهد. پژوهش حاضر با هدف بررسي عوامل ميكروبي شايع در استفاده­كنندگان روش­هاي هورموني و جراحي پيشگيری از بارداری در مراجعين به مراكز بهداشتي درماني شهر همدان انجام شده است. مواد و روش­ها: اين پژوهش يك مطالعه توصيفي تحليلي است. 160 نفر ازمراجعین به مراکز بهداشتی درمانی شهر بر اساس نمونه­گيري سهميه­اي انتخاب شدند. از واحدهاي مورد پژوهش علاوه بر شرح حال و مشخصات دموگرافيك، از ترشحات واژن آنان جهت بررسي لام مرطوب، رنگ­آميزي گرم و كشت، نمونه­برداري انجام شد. روش تجزيه و تحليل داده­ها، آمار توصيفي و استنباطي بود و داده­های به­دست آمده با استفاده از نرم­افزار SPSS مورد آناليز قرار گرفت. نتايج: نتايج پژوهش نشان داد كه اكثريت واحدهاي مورد پژوهش در گروه روش طبيعي (50%) و در روش­هاي جراحي (5/72%) داراي علايم بالینی بودند، در حالي­كه در روش­هاي هورموني (65%)، در استفاده­كنندگان كاندوم (80%) فاقد علايم بالینی واژینیت بودند. يافته­ها نشان مي­دهد كه بين علايم باليني و روش­هاي پيشگيري از بارداري ارتباط معناداری وجود دارد (001/0P=). شيوع تريكوموناس واژيناليس دراستفاده کنندگان روش­هاي جراحي بالاترين شيوع (5%) و در گروه روش­هاي هورموني (5/2%) بوده است، در حالي­كه در استفاده­كنندگان از كاندوم و روش­هاي طبيعي منفي گزارش شده است. گاردنرلا دراستفاده کنندگان روش­هاي جراحي (5/7%)، روش­هاي هورموني (5%) و در روش­هاي كاندوم و طبيعي (5/2%) گزارش شده است. كانديدا آلبيكانس در روش­هاي هورموني (5/7%)، در روش­هاي جراحي و كاندوم (5%) و در روش­هاي طبيعي (5/2%) بوده است. اشريشيا كلي در گروه­هاي كاندوم و هورموني (5/7%) و در روش طبيعي (5/2%) گزارش شده است. نتيجه­گيري: با توجه به شيوع بالاتر تريكوموناس واژيناليس، گاردنرلا، اشريشيا كلي و كانديدا آلبيكانس در روش­هاي پيشگيري از بارداري پيشنهاد مي­گردد آنان تحت پيگيري­هاي مرتب، معاينات منظم و مشاوره قرار گيرند.
